# Case report: Time response of plasma clozapine concentrations on cessation of heavy smoking

**DOI:** 10.3389/fphar.2024.1408915

**Published:** 2024-06-21

**Authors:** Lingyan Qi, Botao Ma, Hongzhen Fan, Siyuan Qi, Fude Yang, Huimei An

**Affiliations:** Peking University, HuiLongGuan Clinical Medical School, Beijing HuiLongGuan Hospital, Beijing, China

**Keywords:** schizophrenia, clozapine, smoking cessation, therapeutic drug monitoring, plasma concentrations

## Abstract

Smoking cessation in patients treated with clozapine might lead to elevated plasma concentrations and severe side effects. This case report investigated the trajectory of clozapine plasma concentrations over time after smoking cessation in a Chinese inpatient with schizophrenia. This case report delineates the temporal response of plasma clozapine concentrations and dose-corrected clozapine plasma concentrations in a 33-year-old inpatient with schizophrenia who had a substantial smoking history and ceased smoking abruptly during dose titration. This case report presents a sudden increase in plasma clozapine concentrations and dose-corrected plasma clozapine concentrations after smoking cessation, followed by a rapid decline in dose-corrected plasma clozapine concentrations during the initial 2 weeks and a return to pre-cessation levels approximately 1 month later. The findings suggest that clinicians and pharmacists should adjust clozapine dosage in accordance with changes in smoking status, taking into consideration the temporal effects. Post-smoking cessation adjustments to clozapine dosage should be coupled with therapeutic drug monitoring, especially for patients with heavy smoking habits. Moreover, the advice of the clinical pharmacist should be considered in complex cases to ensure safe use of clozapine.

## Background

Clozapine (CLO) is a distinctive antipsychotic medication extensively utilized for the management of refractory schizophrenia. Despite its efficacy, the drug is linked to a spectrum of severe adverse effects, including granulocytopenia, gastrointestinal hypofunction, myocarditis, and neurological symptoms. The metabolic pathway of CLO primarily involves the CYP 1A2 enzyme, with additional contributions from various CYP isoforms, namely, CYP 2C9, CYP 2C19, CYP 2D6, and CYP 3A4, all contributing to its biotransformation (Pirmohamed et al., 1995; Olesen and Linnet, 2001; Dragovic et al., 2013). Smoking, a source of polycyclic aromatic hydrocarbons, has the potential to induce CYP1A2 enzyme activity (Zevin and Benowitz, 1999), thereby influencing CLO metabolism. Alterations in smoking habits may consequently impact the pharmacokinetics of CLO. Smoking cessation has been shown to normalize CYP1A2 enzyme levels, resulting in a significant elevation in plasma clozapine concentrations and an associated augmented risk of adverse effects (MacLeod et al., 1997). Given the narrow therapeutic range of clozapine, post-smoking cessation elevation in plasma drug concentrations can lead to clinically significant outcomes (Burns, 1999). In accordance with the Arbeitsgemeinschaft für Neuropsychopharmakologie und Pharmakopsychiatrie (AGNP) consensus guidelines (Hiemke et al., 2018), therapeutic drug monitoring (TDM) for CLO is strongly recommended. Despite this, there is a paucity of studies reporting changes in CLO concentrations following smoking cessation, particularly in Chinese Han patients. The current investigation addresses this gap, presenting the temporal response of plasma CLO concentrations following the cessation of heavy smoking in an inpatient with schizophrenia treated with CLO during the COVID-19 pandemic. The study involving humans was approved by the Review Board of the Beijing Hui-Long-Guan Hospital on 24 October 2019 under registration number 2019-41. The study was conducted in accordance with the local legislation and institutional requirements. The participant provided the written informed consent to participate in this study.

## Case description

The patient under consideration was a 33-year-old unmarried Chinese man with an 8-year history of schizophrenia, displaying poor responsiveness to multiple medication trials. He had no reported family history of psychiatric disorders. Upon hospitalization, the patient presented with hyperlipidemia, hyperuricemia, homocysteinemia, and symptoms indicative of a cold. Additionally, he had a decade-long history of smoking one pack of cigarettes per day. Initial manifestations of the patient’s condition emerged at the age of 25, marked by frequent tantrums, social reluctance, soliloquy, and reduced sleep requirements. The formal diagnosis of schizophrenia occurred at 27 years of age, following the development of delusions, hallucinations, phantom smells, impulsive behaviors, and other psychotic symptoms. Subsequently, over 5 years, the patient underwent six hospitalizations due to severe psychotic symptoms or relapses. Detailed medication information is shown in Table 1. However, satisfactory alleviation of persistent psychiatric symptoms was not achieved.

**TABLE 1:** Medication information for patient from 27 to 32 years of age.

No. of hospitalizations	Age	Medication
1	27 years	Amisulpride, 0.8 g/day
2	28 years	Risperidone, 5 mg/day
3	29 years	Olanzapine, 15 mg/day
4	30 years	Olanzapine, 20 mg/day
5	31 years	Olanzapine, 20 mg/day
6	32 years	Clozapine, 225 mg/day

At the age of 32, the patient was rehospitalized and subjected to CLO treatment. The daily CLO dosage was incrementally elevated from 25 to 225 mg/day, resulting in a corresponding plasma CLO concentrations of 572 ng/mL. Following 3 months of this treatment, the psychotic symptoms exhibited improvement, leading to his discharge from the hospital. No significant adverse reactions were documented during the hospitalization for the first CLO treatment. After discharge, the daily CLO dosage was reduced to 100 mg/mL in the absence of physician consent.

Six months after discharge, the patient was readmitted due to a deterioration in psychotic symptoms. Physical examination revealed no abnormalities. Initial laboratory assessments, including a complete blood count and a basic biochemistry panel, indicated a white blood cell count of 15.31×10^9^/L (reference range 3.5–9.5× 10^9^/L), an absolute neutrophil count of 11.75×10^9^/L (reference range 1.8–6.3× 10^9^/L), an absolute monocyte value of 0.85×10^9^/L (reference range 0.1–0.6×10^9^/L), serum glutamine transaminase of 74 U/L (reference range 9–50 U/L, triglycerides of 2.30 mmol/L (reference range 0–1.7 mmol/L), lipoprotein(a) of 32.7 mg/dL (reference range 0–30 mg/dL), uric acid of 509 μmol/L (reference range 150–440 μmol/L), homocysteine of 47.9 μmol/L (reference range 0–15 μmol/L), and ultrasensitive C-reactive protein (CRP) of 9.35 mg/L (reference range 0.11–3.11 mg/L). After 10 days of hospitalisation, his cold symptoms he had on admission disappeared, and his ultrasensitive C-reactive protein value decreased to 2.9 mg/L, which was within the normal reference range.

After admission, the patient received continuous CLO treatment for his psychotic symptoms. The initial daily dosage was 100 mg/day, escalating to 150 mg/day on day 8, resulting in a plasma CLO concentrations of 342 ng/mL on day 14. Subsequently, the daily dosage was further increased to 200 mg/day on day 15; however, specific information regarding the corresponding plasma CLO concentrations was unavailable. On day 19, the daily dosage was raised to 250 mg/day, leading to a plasma CLO concentrations of 1174 ng/mL on day 23, surpassing the laboratory alert threshold of 1000 ng/mL. On the same day (day 23), smoking were prohibited for the patient due to the closed management policy in response to the COVID-19 outbreak, implemented to prevent cross-infection within the ward. On day 30, the plasma concentrations of CLO was remeasured, yielding a value of 1124 ng/mL, still surpassing the laboratory alert threshold. Consequently, the daily dose was adjusted to 225 mg/day on day 33. On day 37, the plasma CLO concentrations decreased to 821 ng/mL, and no exacerbation of psychosis was observed following the dosage reduction. Subsequent measurements on day 49 and day 57, while maintaining a stable dose of 225 mg/day, recorded plasma CLO concentrations of 721 ng/mL and 590 ng/mL, respectively. The patient was then hospitalized with a consistent CLO dosage of 225 mg/day. No exacerbated psychiatric symptoms or serious adverse events were observed during the month of follow-up after smoking cessation. The patient was discharged after 1 month with significant improvement.

During the first week of hospitalization, the patient received haloperidol and chlorpromazine hydrochloride intramuscularly for agitation. During the first 10 days of the hospitalization, he took Shuanghuanglian oral liquid, an herbal medicine for colds. During his hospitalization, he also took Zhibituo capsules, a Chinese herbal extract for hyperlipidemia. The patient’s medication compliance was checked by the nurses. The CLO was administered twice daily (08:00 and 20:00). No antibiotics were prescribed.

**FIGURE 1:**
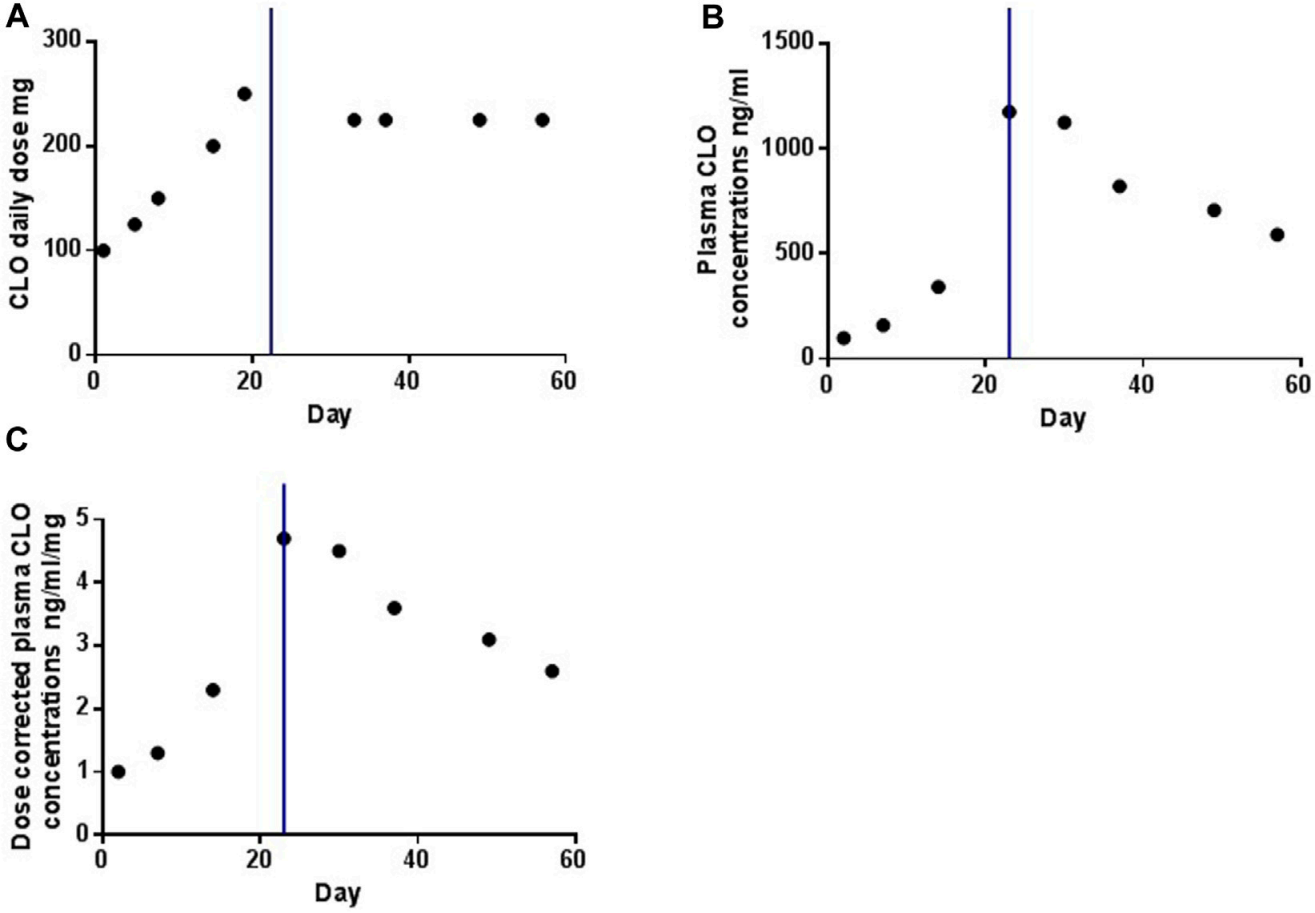
The profiles of daily dose **(A)**, plasma concentrations **(B)** and dose-corrected of plasma concentrations **(C)** of CLO over time. The blue line indicates the time point at which the smoking was ceased.

Figure 1 illustrates the temporal profiles of the daily dose, plasma concentrations, and dose-corrected plasma concentrations of CLO. Following smoking cessation, the plasma CLO concentrations abruptly exceeded the laboratory alert threshold of 1000 ng/mL. Dose-corrected CLO plasma concentrations increased from 2.3 to 4.7 ng/ml/day. In the initial 2 weeks post-smoking cessation, dose-corrected CLO plasma concentrations exhibited a decline from 4.7 to 3.6 ng/ml/day, indicating a reduction rate of approximately 1.67% per day. After 33 days without smoking, the dose-corrected CLO plasma concentrations further decreased to 2.6 ng/ml/day, reflecting a decline rate of approximately 1.38% per day. This value was comparable to the dose-corrected CLO plasma concentrations of 2.5 ng/ml/day observed prior to smoking cessation.

## Discussion

As several previous studies reported, this case report presents that plasma CLO concentrations, as well as dose-corrected plasma CLO concentrations, exhibit a significant increase in patients with schizophrenia who are on stabilized doses after discontinuation of smoking (Derenne and Baldessarini, 2005; de Leon et al., 2020; Tsukahara et al., 2022; Flanagan et al., 2023). For instance, Derenne et al. documented the case of a white female patient, observing an escalation in dose-corrected CLO concentrations from 2.25 to 4.65 ng/mL/day. Following the abrupt cessation of smoking, the patient experienced adverse effects, including sedation, confusion, muscle spasms, dry mouth, dizziness, sluggish pupils, and mild delirium (Derenne and Baldessarini, 2005). Additionally, the case report notes a more rapid decline in dose-corrected plasma CLO concentrations during the initial 2 weeks after smoking cessation, returning to pre-cessation levels approximately 1 month later. This finding could be explained by De Leon’s views, which suggested that the de-induction effect after smoking cessation takes approximately 2–4 weeks due to the need to synthesize new CYP1A2 (de Leon, 2004). Similarly, Faber et al. observed a decrease in CYP1A2 activity by 20% and 36% on days 2 and 7, respectively, after smoking cessation, stabilizing at a new steady state after 1 week (Faber and Fuhr, 2004). Similarly, previous case reports stated that patients with schizophrenia treated with CLO experienced serious adverse events or a sudden increase in plasma CLO concentrations at 2 weeks to 1 month after smoking cessation. For example, Derenne et al. reported the case of a 28-year-old woman with severe psychiatric illness who abruptly stopped her habit of heavy smoking while continuing to take 450 mg of CLO daily. After approximately 4 weeks, she developed serious adverse events. Her serum CLO concentrations exceeded 1000 ng/mL, and these symptoms alleviated rapidly when the CLO dose was reduced (Derenne and Baldessarini, 2005). Bondolfi et al. also reported the case of a 51-year-old man with paranoid schizophrenia who abruptly stopped smoking; 2 weeks later, he started complaining of severe sedation and fatigue with an approximately threefold increase in plasma CLO concentrations (Bondolfi et al., 2005). Based on these studies, it can be speculated that the impact of smoking cessation on CLO metabolism may persist for at least a month, with the most pronounced effects observed in the initial 2 weeks and a gradual diminishment thereafter.

There are some points that need to be mentioned. First, as we all know, infection or inflammation, co-medication with CYP450 inhibitors, and change in dietary or lifestyle can increase plasma CLO concentrations (Clark et al., 2018; Ruan et al., 2018; de Leon et al., 2020). However, in this case, the patient was not taking CYP450 inhibitors, his dietary or lifestyle remained essentially unchanged during hospitalization except for smoking behavior, only the CRP was higher than the normal range during the first 10 days of admission, after which the cold symptoms disappeared and the CRP values dropped to within the normal reference range, therefore, smoking cessation was the main contributor to the increase in CLO concentrations. Second, the patient’s psychiatric symptoms did not worsen after the CLO dose reduction. However, if lower CLO dose was ineffective, several augmentation strategies should be considered, such as the combination of amisulpride, aripiprazole, mirtazapine, omega-3 fatty acids, and electroconvulsive therapy (Porcelli et al., 2012; Grover et al., 2023). Third, CLO is strongly recommended for TDM according to the AGNP consensus guidelines, with a therapeutic reference range of 350–600 ng/mL and a laboratory alert concentration of 1000 ng/mL (Hiemke et al., 2018). In addition, several previous studies have suggested that clinical pharmacist interventions can reduce antipsychotic polypharmacy use and improve treatment guidelines adherence (Stuhec, 2014; Stuhec and Zorjan, 2022; Stuhec et al., 2023). Therefore, in order to improve the safety of CLO administration in clinical practice, dose adjustments should be made in conjunction with TDM data and recommendations from clinical pharmacists.

Several limitations should be considered when interpreting the results of this case report. First, the plasma norclozapine concentrations was not examined. Second, poor CLO metabolism also increased CLO concentrations. However, CYP1A2 genotyping was not performed in this case. Third, no major adverse events were identified in this case. We plan to use the adverse drug reactions probability scale (Naranjo et al., 1981) in future studies to more accurately assess the probability of adverse drug reactions.

This case report delineates the temporal response of plasma CLO concentrations and dose-corrected CLO plasma concentrations in a Chinese Han patient with schizophrenia who had a substantial smoking history and ceased smoking abruptly during dose titration. The findings underscore the importance for clinicians and pharmacists to adjust CLO dosage in accordance with changes in smoking status, taking into consideration the temporal effects. Post-smoking cessation adjustments to CLO dosage should be coupled with TDM, especially for patients with heavy smoking habits. Moreover, the advice of the clinical pharmacist should be emphasized in complex cases to ensure safe CLO use.

## Data Availability

The original contributions presented in the study are included in the article/supplementary material, further inquiries can be directed to the corresponding authors.
